# Dynamic Colloidal Photonic Crystal Hydrogels with Self-Recovery and Injectability

**DOI:** 10.34133/2021/9565402

**Published:** 2021-03-30

**Authors:** Yue Ma, Peiyan He, Wanli Xie, Qiang Zhang, Weiling Yin, Jianming Pan, Miao Wang, Xin Zhao, Guoqing Pan

**Affiliations:** ^1^Institute for Advanced Materials, School of Materials Science and Engineering, Jiangsu University, Zhenjiang, Jiangsu 212013, China; ^2^School of Chemistry and Chemical Engineering, Jiangsu University, Zhenjiang, Jiangsu 212013, China; ^3^Jiangsu Agrochem Laboratory, Changzhou, Jiangsu 213022, China; ^4^Department of Biomedical Engineering, The Hong Kong Polytechnic University, Hung Hom, Hong Kong, China

## Abstract

Simulation of self-recovery and diversity of natural photonic crystal (PC) structures remain great challenges for artificial PC materials. Motivated by the dynamic characteristics of PC nanostructures, here, we present a new strategy for the design of hydrogel-based artificial PC materials with reversible interactions in the periodic nanostructures. The dynamic PC hydrogels, derived from self-assembled microgel colloidal crystals, were tactfully constructed by reversible crosslinking of adjacent microgels in the ordered structure via phenylboronate covalent chemistry. As proof of concept, three types of dynamic colloidal PC hydrogels with different structural colors were prepared. All the hydrogels showed perfect self-healing ability against physical damage. Moreover, dynamic crosslinking within the microgel crystals enabled shear-thinning injection of the PC hydrogels through a syringe (indicating injectability or printability), followed by rapid recovery of the structural colors. In short, in addition to the great significance in biomimicry of self-healing function of natural PC materials, our work provides a facile strategy for the construction of diversified artificial PC materials for different applications such as chem-/biosensing, counterfeit prevention, optical display, and energy conversion.

## 1. Introduction

Artificial photonic crystal (PC) materials mimicking the naturally occurring periodic nanostructures in creatures like a chameleon or a butterfly have attracted increasing interest [1–10]. These bioinspired materials have shown great potentials in chem-/biosensing [11–16], bioelectronics [15, 17–19], optical display [20–24], energy conversion [7, 25–27], and others [28–35] due to their unique properties such as brilliant and stable structural colors, slow light, and amplified photon absorption/emission. Although great achievements in artificial PC materials have been made in mimicking the photonic nanoarchitectures in nature, the functional simulation is an area still in its infancy. For instance, the creatures can spontaneously heal from injury and recover their functionality to increase the survivability and lifetime. The simulation of such self-healing property is of critical importance to the PC materials, since accidental cuts and scratches are inevitable during applications, which may deteriorate the performance of these materials. Therefore, artificial PC materials with diversified and complex nanostructures as well as self-recovery capability and mechanical durability are highly sought after.

Amongst various PC materials, PC hydrogels feature the best flexibility and plasticity, considering the fragility of other hard structure-color materials. To import periodic nanostructures in hydrogel networks during fabrication of PC hydrogels, colloidal crystal templating methods were initially employed [36]. After filling, gelling, and etching of the ordered assemblies of hard spheres like silica dioxide, poly(methyl methacrylate), and polystyrene nanoparticles, inverse opal-like PC hydrogels with various structure colors could be obtained [12, 37–39]. Alternatively, PC hydrogels could be prepared by covalent crosslinking of microgel colloidal crystals [40–43]. In contrast to the poor controllability of hard colloidal crystal templating strategy, the postcrosslinking method towards soft microgels enables large-scale and fast production of PC hydrogels. Nevertheless, the above two strategies both involve an irreversible solidifying process of the periodic nanostructures that can be permanently damaged by external mechanical stress. In this regard, self-healing hydrogels with nonsupporting inverse opal structures [44] or injectable hydrogels incorporated with photonic supraballs [45] were prepared. These designs indeed endow PC hydrogels with improved survivability or operability. They, however, lack the internally dynamic characteristic of natural PC materials (e.g., self-recovery of biogenic periodic nanostructures on the damaged interfaces after injury) [4, 6]. Besides, the self-healing mechanisms in these systems mainly relied on the formation of hydrogen bonds, van der Waals forces, or host-guest interactions between the filled hydrogel matrix, whose strength was relatively low for the healing of fracture interface and the maintenance of stable periodic nanostructures [46, 47].

**Scheme 1:**
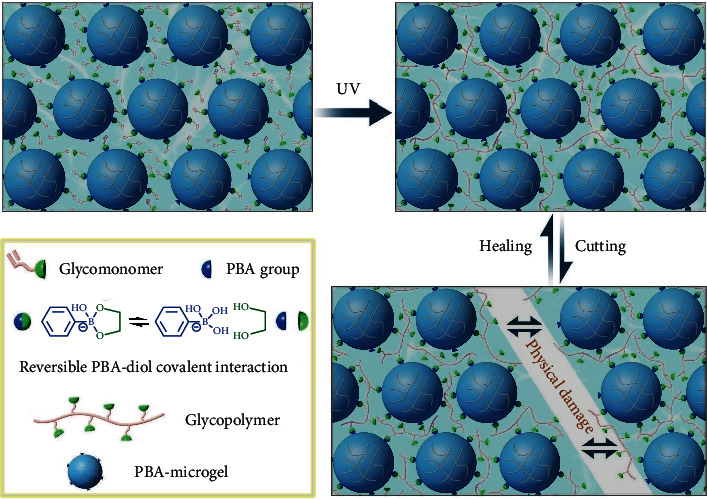
Schematic illustration of the reversible crosslinking strategy and self-recovery mechanism of the proposed dynamic colloidal PC hydrogels based on dynamic phenylboronate covalent bond.

To obtain the internally dynamic PC hydrogels, we shift our focus to the interaction forces of the particle assembly within the microgel colloidal crystals. Although covalent postcrosslinking of adjacent particles can increase their stability to form a colloidal PC hydrogel, the dynamic nature of noncovalently assembled microgel colloidal crystals will be lost. To balance the stability and the dynamics in a colloidal PC hydrogel, here, we propose to use reversible covalent interactions to dynamically stabilize the assembled nanoparticles in microgel colloidal crystals. Such reversible but strong covalent interactions will be achieved by using phenylboronic acid (PBA) derivatives and *cis*-diols (boron-carbohydrate interaction, Scheme 1) [48–50]. In this context, we intend to prepare microgels with PBA groups and then place them in a glycomonomer solution to self-assemble into ordered colloidal crystals. As PBA is capable of reversible interaction with the *cis*-diol groups of glycomonomers, photoinitiated polymerization of the glycomonomer will lead to dynamic crosslinking of adjacent microgel particles though reversible PBA-*cis*-diol interactions between the microgels and the resultant glycopolymers. Such unique dynamic interaction between the particles and the matrix enables the reconfiguration of the assembly units during the self-healing process rather than merely the recombination of the filled hydrogel matrix. In this case, an injectable and self-healing PC hydrogel formed upon dynamic covalently assembled microgel photonic crystals can be obtained. We envision that our dynamic colloidal PC hydrogels will show great potential in constructing diversified and complicated PC materials for novel applications in chem-/biosensing, counterfeit prevention, optical display, and energy conversion.

## 2. Results and Discussion

**Figure 1:**
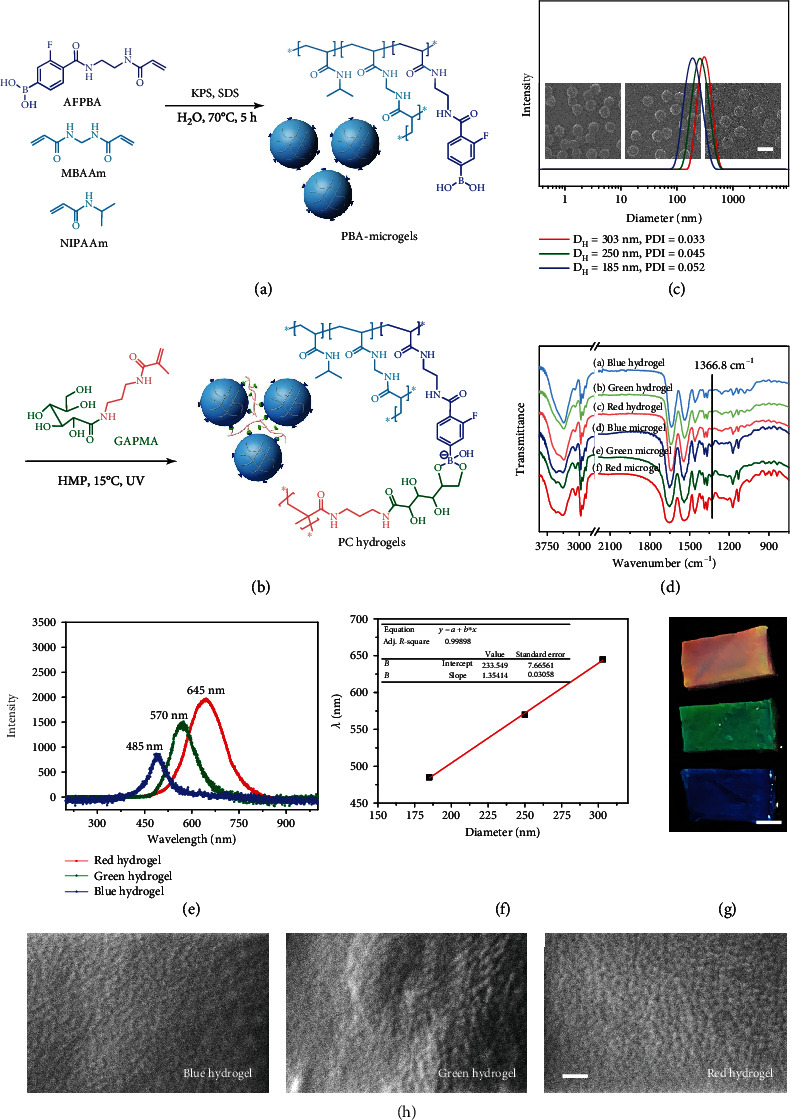
(a) Synthesis of the PBA-microgels. (b) Dynamic crosslinking of the preassembled PBA-microgel colloidal crystals via photoinitiated polymerization of *cis*-diol-containing glycomonomers. (c) Dynamic light scattering (DLS) analysis of the PBA-microgels. Inserts: SEM images showing the morphology of the dry PBA-microgels (average diameters of the PBA-microgels in corresponding SEM images from left to right were 130, 160, and 190 nm). Scale bar = 200 nm. (d) FT-IR spectra of the PBA-microgels and PC hydrogels. The reaction between PBA and *cis*-diol groups in GMAPMA resulted in a weakened peak of B-O stretching (1366.8 cm^−1^). (e) Reflection spectra and the corresponding photos of three typical colloidal PC hydrogels. (f) The linear relation between the diameter of PBA-microgels and photonic stopband of the corresponding colloidal PC hydrogels. The reflection peak (*λ*) of PC materials can be estimated by Bragg's equation, *λ* = 2*d*_111_*n*_average_, in which *d*_111_ is the interplanar distance of (111) diffracting planes and *n*_average_ is the average refractive index of the materials. (g) Photos of the three PC hydrogels. Hydrogel sizes: 1.5 cm × 3.0 cm. Scale bar = 1 cm. (h) SEM images of the lyophilized hydrogels with short-range order in the assembled microgels. Scale bar is 500 nm.

According to our previous method [51], PBA-containing microgels (PBA-microgels) were synthesized in aqueous solution through surfactant-assistant precipitation polymerization, using sodium dodecyl sulfate (SDS), potassium persulfate (KPS), 4-(2-acrylamidoethylcarbamoyl)-3-fluorophenylboronic acid (AFPBA), *N*-isopropylacrylamide (NIPAAm), and methylene bisacrylamide (MBAAm) as the surfactant, initiator, PBA functional monomer, backbone monomer, and the crosslinker, respectively (Figure 1(a)). SDS was chosen because it could regulate the sizes of microgels [52], while AFPBA was selected and synthesized as it could reversibly interact with *cis*-diols (Figure S1) [48]. To achieve dynamic crosslinking in the microgel colloidal crystals, the resultant PBA-microgels were first coincubated with a glucose-derived glycomonomer 3-gluconamidopropyl methacrylamide (GAPMA, Figure S2) and a water-soluble photoinitiator (2-hydroxyethoxy)-2-methylpropiophenone (HMP). UV light-initiated polymerization of GAPMA monomers was then switched on when the PBA-microgels self-assembled to ordered structures and showed clear structural colors. Finally, the ordered PBA-microgels formed dynamic colloidal PC hydrogels due to the reversible covalent interactions between the PBA-microgels and the poly(GAPMA) glycopolymers (Figure 1(b)).

To obtain colloidal PC hydrogels with different structural colors, three types of PBA-microgels with different sizes were prepared by regulating the concentrations of SDS and MBAAm (Figure 1(c)). Dynamic light scattering (DLS) analysis indicated that all three microgels showed uniform sizes with diameters increasing from 185, 250 to 303 nm. Scanning electron microscopy (SEM) images revealed that all the microgels exhibited spherical shape. It should be noted that, although the sizes of microgels reflected from DLS and SEM (wet vs. dry) were different, an increasing trend in the diameters could still be observed. Chemical components of the microgels were further characterized using infrared spectroscopy (IR) (Figure 1(d)). In addition to the characteristics of NIPAAm units [53], a characteristic peak of AFPBA (B-O stretching) [54] at 1366.8 cm^−1^ was also observed in all the spectra. These results indicated the successful preparation of size-different but monodispersed PBA-microgels.

The PBA-microgels were then self-assembled into colloidal photonic crystals in aqueous solution with the glycomonomer GAPMA, following by UV light-initiated polymerization (Figure 1(b)). The weakened peaks of B-O stretching at 1366.8 cm^−1^ in the IR spectra of three hydrogels confirmed the efficient formation of phenylboronate bonds between PBA and the *cis*-diols of GAPMA (Figure 1(d)) [55]. As expected, photoinduced crosslinking of the PBA-microgels with GAPMA efficiently led to the gelation of the systems. As shown in Figures 1(e) and 1(g), three colloidal PC hydrogels with blue (485 nm), green (570 nm), and red (645 nm) structural colors were obtained from the PBA-microgels with diameters of 185, 250, and 303 nm, respectively. Further study revealed a linear correlation between the microgel sizes and the photonic stop band wavelengths (Figure 1(f)), which was in good agreement with Bragg's law. We then further used SEM to check the assembled microgels in the PC hydrogels (Figure 1(h)). Short-range order could be clearly observed in the arrangement of all three hydrogels, which contributed mainly to the colors reflected on the PC hydrogels. These results together demonstrated that our PBA-based dynamic crosslinking strategy could be used as a general method for the construction of stable colloidal PC hydrogels with diverse structural colors.

**Figure 2:**
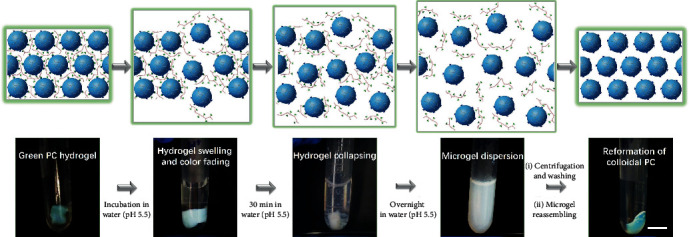
Investigation of the internally dynamic characteristics of phenylboronate-crosslinked colloidal PC hydrogel through an acid-triggered disassembly and reassembly method. Scale bar = 1 cm.

Unlike previous PC hydrogels with fixed crystal structures [40–45], reversible phenylboronate-crosslinked strategy would lead to dynamic colloidal PC hydrogels with mobilizable periodic nanostructures. To demonstrate the internally dynamic characteristic of our colloidal PC hydrogel, an acid-induced disassembly and reassembly experiment was carried out (Figure 2). In view of the pH-reversibility of PBA-*cis*-diol interactions, our phenylboronate-crosslinked colloidal PC hydrogels were found able to disassemble and reassemble by changing the pH values. As expected, when a piece of green colloidal PC hydrogel was incubated in an acidic aqueous solution (pH 5.5), rapid collapsing of the PC hydrogel and subsequently complete dispersion of the microgels into the solution were observed (milky white solution). This is attributed to the reversible crosslinking of the assembled microgels, which enabled acid-induced decrosslinking of the colloidal hydrogels. Moreover, the dispersed microgels could reassemble into colloidal crystals with a green structural color at pH 7.5. These results confirmed that the phenylboronate-crosslinked colloidal PC hydrogels, similar to the natural PC materials, boast the reversibility in the periodic nanostructures, suggesting the potential of self-recovery in practical applications like colorimetric sensors, wearable optical devices, and anticounterfeiting labels.

**Figure 3:**
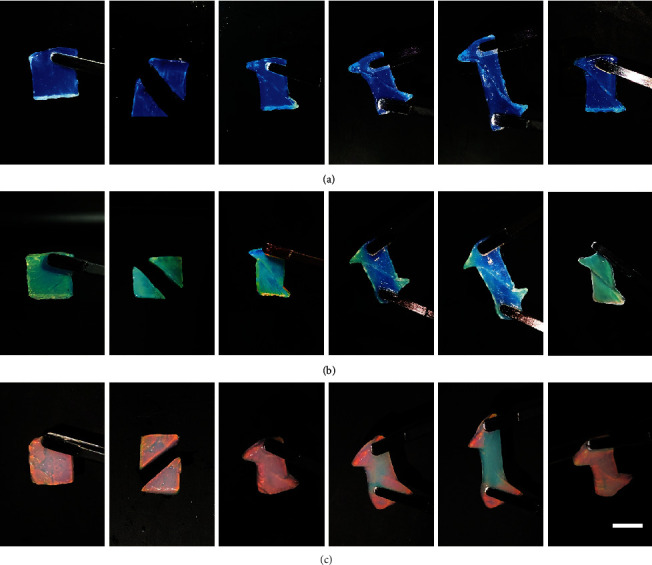
Visual demonstration of self-healing properties of the three colloidal PC hydrogels with PBA-microgels at different diameters against physical damage. Hydrogel sizes: 1.5 cm × 1.5 cm. (a) Blue PC hydrogel; (b) green PC hydrogel; (c) red PC hydrogel. Scale bar = 1 cm.

The dynamic and self-recovery of the three colloidal PC hydrogels were first examined by investigating their self-healing ability against physical damage (Figure 3 and S3). The blue, green, and red hydrogels were all cut into two halves, and then the two halves were staggered and contacted for self-healing. All the colloidal PC hydrogels showed very fast healing at the interfaces of damage within seconds, and no breakage was found even when the healed hydrogels were subjected to a stretching force. In addition, no significant difference was observed on the reflection spectra of the repaired sections when the stretching force was removed. Interestingly, we found that the external tension applied on the colloidal PC hydrogels all led to a blue shift in the structural colors (Figure 3), mainly due to the decreased interplanar distance of the diffracting plane under tension, indicating one of the potential applications of our PC hydrogels as a mechanical sensor [44]. Nevertheless, the hydrogels could recover their original structural colors once the tension forces were removed and the hydrogels shrunk to their original sizes. In addition, we found our colloidal PC hydrogels could be stretched up to 155% of the original length, indicating good flexibility to resist an external tensile force (Figure S4). These phenomena indicated that our PC hydrogels possessed self-reparability and reversibility in the structural colors.

**Figure 4:**
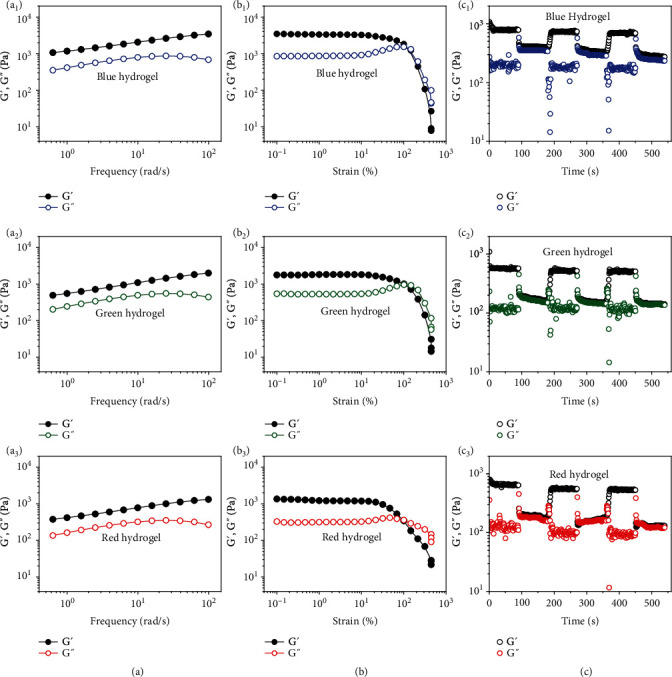
(a) Dynamic oscillatory frequency sweeps (*γ* = 1%), (b) strain amplitude sweeps (*ω* = 1 rad s^−1^), and (c) step-strain sweeps (*γ* = 1% or 150%, *ω* = 1 rad s^−1^) of the dynamic colloidal PC hydrogels at 15°C. G′: storage modulus; G^″^: loss modulus.

To further demonstrate the self-recovery, rheology examination of the colloidal PC hydrogels was carried out. Dynamic oscillatory frequency sweeps were first studied with frequencies (*ω*) ranging from 0.1 to 100 rad s^−1^ (Figure 4(a)). All the hydrogels showed frequency dependence viscoelastic behaviors with a higher storage modulus (G′) than the loss modulus (G^″^) across the whole range of frequencies tested, suggesting their dynamic and gel-like characteristics [56, 57]. Strain sweep tests were then performed to find the critical strain values required to damage the gel networks. As shown in Figure 4(b), the G′ and G^″^ of all three hydrogels kept constant until the strain (*γ*) reached up to 100%, indicating good resistance for mechanical agitations. However, a dramatic decrease was observed for both G′ and G′′ when the strain was further increased. This is resulted from the severe breakage of dynamic PBA-*cis*-diol interactions and collapse of the hydrogel networks, showing a transition of the colloidal PC hydrogels from gel to sol state [49]. The self-recovery of the hydrogels after network failure under high strains was finally investigated using a step-strain sweep with alternate low (1%) and high (150%) strains. As shown in Figure 4(c), the mechanical properties of all three colloidal PC hydrogels could immediately recover as soon as the highoscillation excitation was removed. Moreover, repeated dynamic strain sweep revealed that both G′ and G^″^ could get back to their initial values without significant loss, demonstrating the robust self-healing property of our PC hydrogels and fast recovery of the hydrogel networks after physical damage. This result, together with the self-reparability of structural colors, jointly demonstrated the perfect self-recovery of our PC hydrogels in both structure and function.

**Figure 5:**
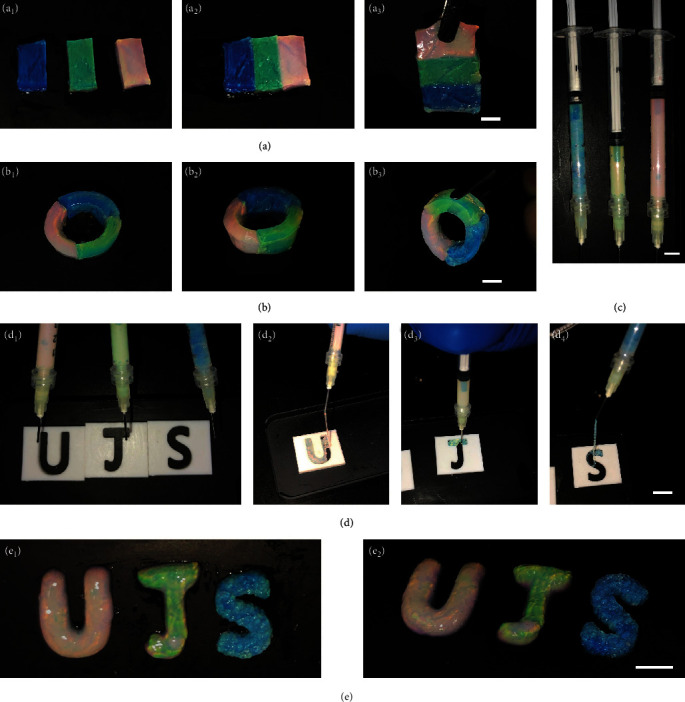
(a) The assembled hybrid colloidal PC hydrogels with three combinatorial structure colors at 2D plane. Hydrogel sizes: 1.5 cm × 3.0 cm. Scale bar = 1 cm. (b) The assembled 3D curved colloidal PC hydrogel ring. Scale bar = 1 cm. (c) The three dynamic colloidal PC hydrogels could be easily transferred into syringes. Scale bar = 1 cm. (d) The injection of dynamic colloidal PC hydrogels for the construction of photonic letter patterns. Teflon molds: 3.0 cm × 3.0 cm. Scale bar = 1 cm. (e) The photos of photonic letter patterns shot from different angles (90° and 45°). Scale bar = 1 cm.

In addition to self-recovery, another remarkable characteristic of PC hydrogels is the diversity of coloration derived from combinatorial structural colors. To demonstrate the versatility of our self-healing colloidal PC hydrogels for mimicking the multistructural colors of natural PC materials, three pieces of hydrogels with different colors were used for conceptual construction of artificial PC materials with combinatorial structural colors (Figure 5). Similar to the self-healing mechanism through the formation of interfacial PBA-*cis*-diol interactions, the red, green, and blue colloidal PC hydrogels could easily assemble together with stable mechanical strength, resulting in a hybrid colloidal PC hydrogel displaying three combinatorial structural colors (Figure 5(a)). In addition to the multistructural assembly in the 2D plane, the PC hydrogels were also used to construct a 3D curved hydrogel ring. As shown in Figure 5(b), a three-color PC hydrogel ring could be readily obtained, implying the possibility of our dynamic colloidal PC hydrogels for precise construction of PC devices with complex macrostructures and multiple structural colors.

Apart from the versatility in multistructural assembly, the mechanical agitation-induced gel-to-sol transition also implied the possibility of our colloidal PC hydrogels for injection. Since the disruption of dynamic crosslinks by shear stress would provide broken segments with higher mobility within the hydrogel matrix, the shear-thinning behaviors were visually demonstrated by injecting our colloidal PC hydrogels through a needle (21 G). The hydrogels could be easily placed into a syringe and then applied with pressure for injection. As shown in Figures 5(c) and 5(d) and Movie S1, all the hydrogels showed bright structural color due to the formation of ordered structure via reversible phenylboronate covalent bond before injection. These hydrogels could be favorably injected through the syringe followed by a rapid recovery of the structural colors. By using predesigned Teflon molds with letter patterns, three hydrogel letters with different structural colors could be constructed (Figure 5(e)). In addition, the three injected PC hydrogel letters all showed bright colors from different angels, i.e., angle-independent structural colors. Next, we performed the extrusion-based 3D printing test to demonstrate the printability of our colloidal PC hydrogels. As shown in Figure S5, a multilayered 3D scaffold with a fiber diameter of ~500 *μ*m was obtained using blue and green PC hydrogels as the inks, which exhibited high printing resolution. Conceivably, the injectability, moldability, and printability of our dynamic PC hydrogels reflected a very attractive potential in the development of diversified artificial PC materials or scaffolds for different biomedical applications.

## 3. Conclusion

In summary, we propose here a reversible boronate covalent strategy for the design of dynamic colloidal PC hydrogels with self-healing and injectable properties. The motivation of this study is to structurally and functionally mimic the dynamic characteristic and complexity of natural PC materials (e.g., self-recovery and diversity of biogenic periodic nanostructures.). Through reversible crosslinking of adjacent particles in self-assembled microgel colloidal crystals, the dynamic colloidal PC hydrogels could be tactfully constructed. As proof of concept, three types of hydrogels with different microgel sizes and structural colors were prepared in this work. Due to the reversible covalent interactions within the microgel colloidal crystals, the resultant PC hydrogels showed perfect self-healing ability against physical damage and rapid recovery of the structural colors after injection. These properties suggested the superiority of our dynamic colloidal PC hydrogels for the construction of artificial PC materials with complex macrostructures, multiple structural colors, and strong mechanical stress tolerance. We believe that our dynamic colloidal PC hydrogels will be highly promising for different applications, such as environment monitoring, anticounterfeiting, optical devices, energy conversion, and biomedical engineering. Overall, in addition to the great significance in functional biomimicry of natural PC materials, our work also presents an available easy-to-perform strategy for the development of diversified artificial PC materials or devices.

## 4. Materials and Methods

### 4.1. Materials

The solvents deuterium oxide (D_2_O) and dimethyl sulfoxide-d_6_ (DMSO-d_6_) (99.5 atom % D, Sigma-Aldrich) for ^1^H NMR spectroscopy, *N*-isopropylacrylamide (NIPAAm, 98%, Macklin), crosslinker *N*,*N*′-methylenebis(acrylamide) (BIS, 99%, Aladdin), 4-carboxy-3-fluorophenylboronic acid (CFPBA, 98%, J&K), sodium dodecyl sulfate (SDS, 98%, Aladdin), photo initiator 2-hydroxy-4′-(2-hydroxyethoxy)-2-methylpropiophenone (HMP, 98%, Aladdin), and potassium persulfate (KPS, 99.5%, Sinopharm) were used as received. Dichloromethane (DCM), triethylamine (TEA), tetrahydrofuran (THF), and *N*,*N*-dimethylformamide (DMF) for chemical synthesis were dried with CaH_2_ and distilled by a general method before use. Other reagents and AR-grade solvents in this work were purchased from Shanghai Reagent General Factory and used as received. Milli-Q water was purified with a Thermo Scientific Barnstead NANOpure Diamond Water Purification Systems to give a minimum resistivity of 18.2 M*Ω*·cm. The phenylboronic acid (PBA) monomer 4-(2-acrylamidoethylcarbamoyl)-3-fluorophenylboronic acid (AFPBA) and glycomonomer 3-gluconamidopropyl methacrylamide (GAPMA) were synthesized as follows.

### 4.2. Synthesis of Phenylboronic Acid Monomer (AFPBA)

The phenylboronic acid monomer 4-(2-acrylamidoethylcarbamoyl)-3-fluorophenylboronic acid (AFPBA) was synthesized according to a previously reported method [51]. 4-Carbamoyl-3-fluorophenylboronic acid (CFPBA) (5 g, 27.2 mmol) was added to 50 mL of thionyl chloride and refluxed for 6 h under an argon atmosphere. After evaporation in vacuum, the residual solid was mixed with N-carboxybenzoxy-1,2-diaminoethane hydrochloride (9.4 g, 40.8 mmol) in THF (90 mL), to which 28 mL of trimethylamine was slowly added in an ice-cooled bath, stirring at ambient temperature for 6 h. The resulting solution was washed with diluted hydrochloric acid brine, and the organic layer was evaporated to obtain a white solid. It was then dissolved in 400 mL of ethanol and was hydrogenized in the presence of 10% Pd/C (1.0 g) at 40°C for 3 h. The mixture was then filtrated and evaporated using a rotary evaporator. The obtained white solid was dried and dissolved in 150 mL of sodium carbonate aqueous solution (100 mM, pH 10). Acryloyl chloride (4.0 mL, 50 mmol) was added in an ice-cooled bath at weakly basic pH using NaOH aqueous solution. After stirring at ambient temperature for 6 h, the mixture was acidified using HCl solution, and the resulting AFPBA was recrystallized in acetone. The obtained crystal was filtrated, washed with acetone, and finally left to dry in a vacuum. (45% in yield): the chemical structure was characterized by ^1^H NMR and LC-MS.

^1^H NMR (300 MHz, d6-DMSO, Figure S1): *δ* 8.37 (s, 2H, B(OH)_2_); *δ* 8.23~8.24 (m, 1H, -CO-NH-); *δ* 7.55~7.65 (3H, Ar-H); *δ* 6.07~6.21 (m, 2H, -CH=CH_2_); *δ* 5.58~5.60 (d, 1H, -CH=CH_2_); *δ* 2.30~3.33 (m, 4H, -NHCH_2_CH_2_NH-); LC-MS (Figure S1): 280.32 [M]; 281.31 [M+H]^+^; 303.14 [M+Na]^+^.

### 4.3. Synthesis of the Glycomonomer (GAPMA)

GAPMA glycomonomer was prepared according to a previous method. D-gluconolactone (20 g, 112.3 mmol) and APMA (25 g, 140.4 mmol) were dissolved in 400 mL methanol. Then, the solution was added with triethylamine (20 mL, 142.8 mmol). The mixture was stirred overnight at 25°C. The resulting mixture was then filtered with a G5 funnel and then concentrated under vacuum and precipitated out in methanol at -20°C. The crude product was dissolved in DMF and recovered by a large excess amount of acetone. The obtained white solid was dried in vacuo (23.3 g, 65.4% in yield): the chemical structure was characterized by ^1^H NMR.

^1^H NMR (400 MHz, D2O, Figure S2) *δ* 5.62 (s, 1H), *δ* 5.37 (s, 1H), *δ* 4.24~4.23 (d, 1H), *δ* 4.01 (t, 1H),*δ* 3.54~3.75 (m, 4H), *δ* 3.24~3.21 (t, 4H), *δ* 1.85 (s, 3H), *δ* 1.68~1.72 (q, 2H).

### 4.4. Preparation of PBA-Containing Microgels (PBA-Microgels)

The typical procedure for the synthesis of the phenylboronic acid-containing microgels (PBA-microgel-S) is presented as follows: NIPAAm (740 mg), BIS (22 mg), and SDS (12 mg) were added into a three-neck round-bottom flask (100 mL) and then 50 mL of deionized water was put into the flask. A clear solution was obtained after ultrasonication and purged with N_2_ for 30 min at room temperature. Then, the flask was immersed into a thermostatic water bath at 35°C with N_2_ purged continually. AFPBA (78 mg, dissolved in 0.5 mL of DMF) was transferred to the flask. After the solution was heated to 70°C, KPS (40 mg, dissolved in 2.5 mL of deionized water) was dropped to initiate the reaction. The polymerization lasted for 5 h at 70 with N_2_ condition. After the reaction, the white suspension was purified by dialysis against water with frequent water change for 2 weeks, lyophilized, and stored in a refrigerator for further use (entry 1 in Table S1).

PBA-microgel-M and PBA-microgel-L were synthesized and purified under the identical conditions except for changing the ratio of the reagents (entries 2 and 3 in Table S1).

### 4.5. Preparation of Dynamic Colloid Photonic Crystal Hydrogels

The typical procedure for the synthesis of dynamic colloidal PC hydrogel (blue hydrogel) is presented as follows: photoinitiator HHMP (6.6 mg), glycomonomer GAPMA (40 mg), and crosslinker BIS (2 mg) were dissolved in 1 mL of water solution (pH 7.5). A clear solution was obtained after ultrasonication, and then the PBA-microgel-S (128 mg) was transferred to the monomer solution. After swelling 12 h in the dark, a blue colloid crystal was obtained, and then it was transferred into a 10 × 10 × 3 mm mold. The system was then photopolymerized by exposure to UV light at 15°C for 24 h (*λ* = 365 nm). The resulting films were released from the device and stored in a hermetical vessel at 4°C for further use (entry 4 in Table S2).

The green and red PC hydrogels (green hydrogel and red hydrogel) were synthesized similarly (entries 5 and 6 in Table S2).

### 4.6. Characterization

The structures of two synthesized monomers (AFPBA and GAPMA) were measured by ^1^H NMR (Varian Unity plus-400 spectrometer, 400 MHz) using D_2_O or DMSO as solvent. The morphologies of the dry microgels were characterized with a SEM. DLS study was performed to obtain their sizes and particle dispersion indices (PDIs) in water. A DLS spectrometer (ZETAPALS/BI-200SM) with a detector (CROSS-PMT) was utilized for this purpose. The scattered light of a vertically polarized He-Ne laser (659 nm) was measured at an angle of 90° at room temperature. The compositions of all the nanoparticles and color colloid crystals were obtained by Fourier transform infrared spectroscopy (FT-IR, Nicolet Magna-560 FTI spectrometer). Reflection spectra of the PC materials were measured with a fiber optic spectrometer (AvaSpec-2048). Rheology examination of the colloidal PC hydrogels was carried out on an Anton Paar MCR301 rheometer at 15°C with a 25 mm diameter flat plate attached to a transducer. In addition, we performed tensile tests to characterize the mechanical properties of the green PC hydrogel through a universal mechanical testing system (Instron, US). Specimens with dimensions of 20 × 5 × 1.5 mm (length × width × height) were loaded onto the testing machine, and the tensile test was performed at room temperature with a speed of 1 mm/min until specimen failure. We also evaluated the 3D printability of our dynamic PC hydrogels. The pregel solution of the PC hydrogel was printed by extrusion-based 3D printer (Panowin F1) through a 20 G plastic-steel flat tip needle with the aid of 3DMax software. The printing pump was set at 4°C and a moving speed of 0.35 mm/s.

## Data Availability

The data that support the findings of this study are available from the corresponding author on request.

## Supplementary Materials

Supplementary 1 Movie S1: the injectability of dynamic colloidal photonic hydrogels.

Supplementary 2 Table S1: the ratio of NIPAAm, BIS, SDS, and AMCFPBA in the precipitation polymerization. Table S2: the parameters for PC hydrogel construction. Figure S1: 1H NMR and ESI-MS of 4-(2-acrylamidoethylcarbamoyl)-3-fluorophenylboronic acid (AFPBA). Figure S2: 1H NMR spectra for 3-gluconamidopropyl methacrylamide (GAPMA) monomer in D2O. Figure S3: the enlarged images of self-healing process of the colloidal PC hydrogels. Figure S4: the stress-strain curve of tensile test of green colloidal PC hydrogel. Figure S5: 3D printability of the blue and green PC hydrogels (scale bar = 5 mm).
