# Supplementary material for: Dynamic Colloidal Photonic Crystal Hydrogels with Self-Recovery and Injectability
Source: Research (Wash D C). 2021 Mar 30;2021:9565402. doi: 10.34133/2021/9565402 (PMC8028842; doi:10.34133/2021/9565402)
Supplement: Supplementary 2 — Table S1: the ratio of NIPAAm, BIS, SDS, and AMCFPBA in the precipitation polymerization. Table S2: the parameters for PC hydrogel construction. Figure S1: 1H NMR and ESI-MS of 4-(2-acrylamidoethylcarbamoyl)-3-fluorophenylboronic acid (AFPBA). Figure S2: 1H NMR spectra for 3-gluconamidopropyl methacrylamide (GAPMA) monomer in D2O. Figure S3: the enlarged images of self-healing process of the colloidal PC hydrogels. Figure S4: the stress-strain curve of tensile test of green colloidal PC hydrogel. Figure S5: 3D printability of the blue and green PC hydrogels (scale bar = 5 mm). [file 9565402.f2.docx]

Dynamic Colloidal Photonic Crystal Hydrogels with Self-Recovery and Injectability

*Yue Ma,^1,2^ Peiyan He,^2^* *Wanli Xie,^1,2^ Qiang Zhang,^3^ Weiling Yin,^1^ Jianming Pan,^2^ Miao Wang,^1^ Xin Zhao^3,^* and Guoqing Pan^1,^**

^1^ Institute for Advanced Materials, School of Materials Science and Engineering, Jiangsu University, Zhenjiang, Jiangsu 212013, China.

^2^ School of Chemistry and Chemical Engineering, Jiangsu University, Zhenjiang, Jiangsu 212013, China

^3^ Department of Biomedical Engineering, The Hong Kong Polytechnic University, Hung Hom, Hong Kong, China

* Correspondence should be addressed to Xin Zhao; [xin.zhao@polyu.edu.hk](mailto:xin.zhao@polyu.edu.hk) and Guoqing Pan; [panguoqing@ujs.edu.cn](mailto:panguoqing@ujs.edu.cn)

**Table S1.** The ratio of NIPAAm, BIS, SDS and AMCFPBA in the precipitation polymerization.

| **Entry** | **Designation** | **NIPAAm** | **BIS** | **SDS** | **AFPBA** | **Size (nm)^1^** |
| --- | --- | --- | --- | --- | --- | --- |
| 1 | PBA-microgel-S | 740 mg | 22 mg | 12 mg | 78 mg | 185 |
| 2 | PBA-microgel-M | 740 mg | 22 mg | 6 mg | 78 mg | 250 |
| 3 | PBA-microgel-L | 890 mg | 26 mg | 6 mg | 94 mg | 300 |

^1^ The sizes were characterized by DLS.

**Table S2.** The parameters for PC hydrogel construction.

| **Entry** | **Designation** | **GAPMA** | **BIS** | **PBA-NP** | **HHMP** |
| --- | --- | --- | --- | --- | --- |
| 4 | blue hydrogel | 40 mg | 2 mg | 128 mg | 6.6 mg |
| 5 | green hydrogel | 40 mg | 1.5 mg | 128 mg | 6.6 mg |
| 6 | red hydrogel | 40 mg | 1 mg | 128 mg | 6.6 mg |


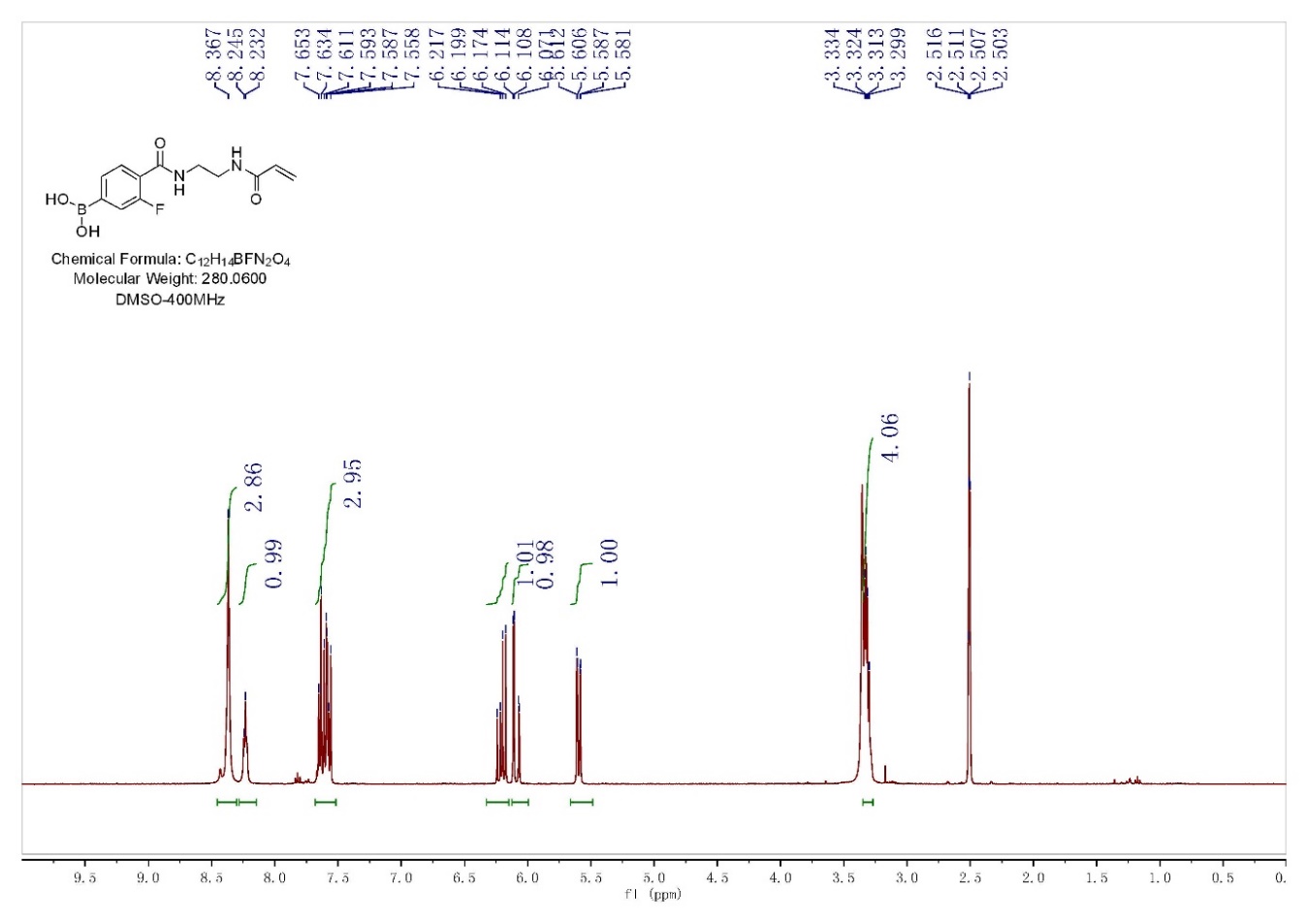


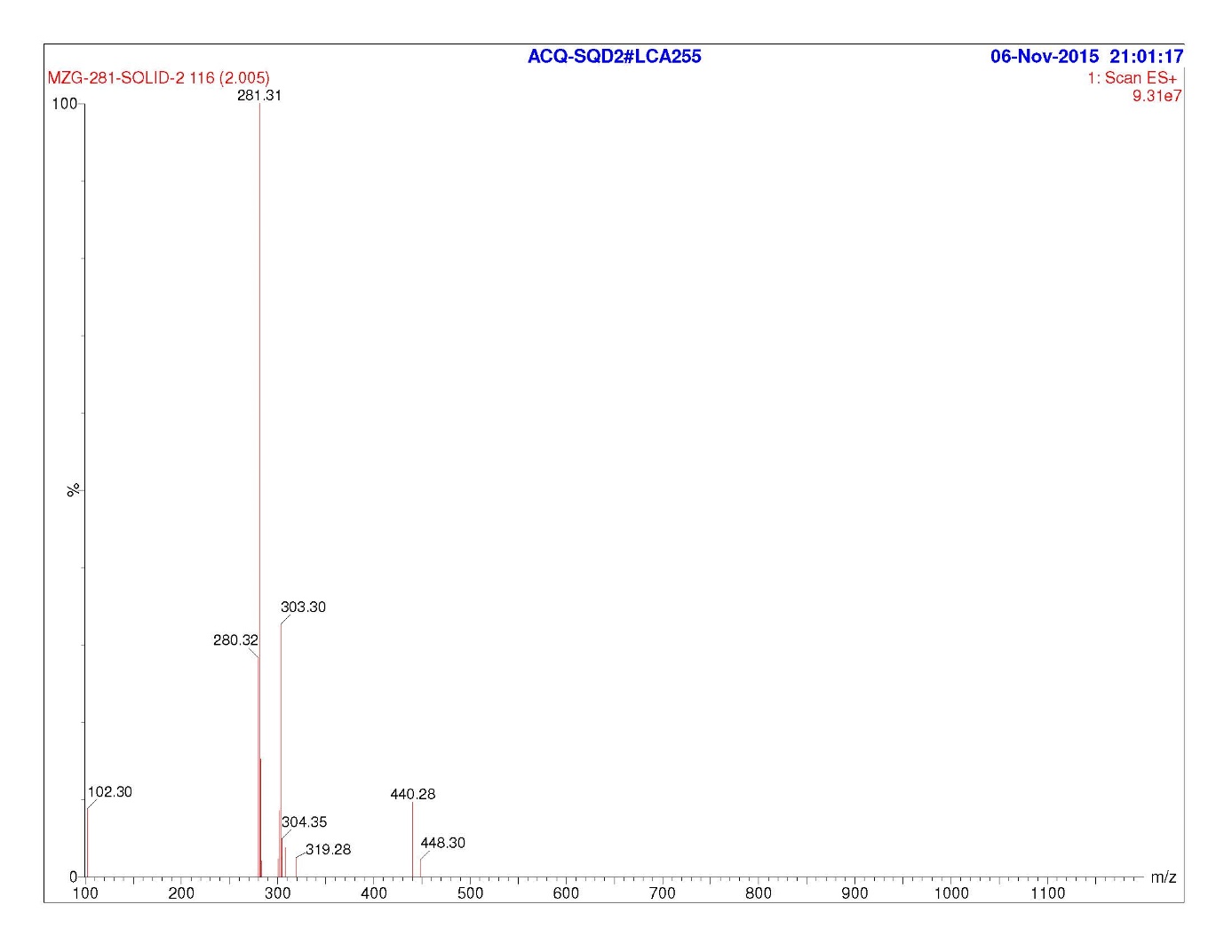


**Figure S1.** ^1^H NMR and ESI-MS of 4-(2-acrylamidoethylcarbamoyl)-3-fluorophenylboronic acid (AFPBA).


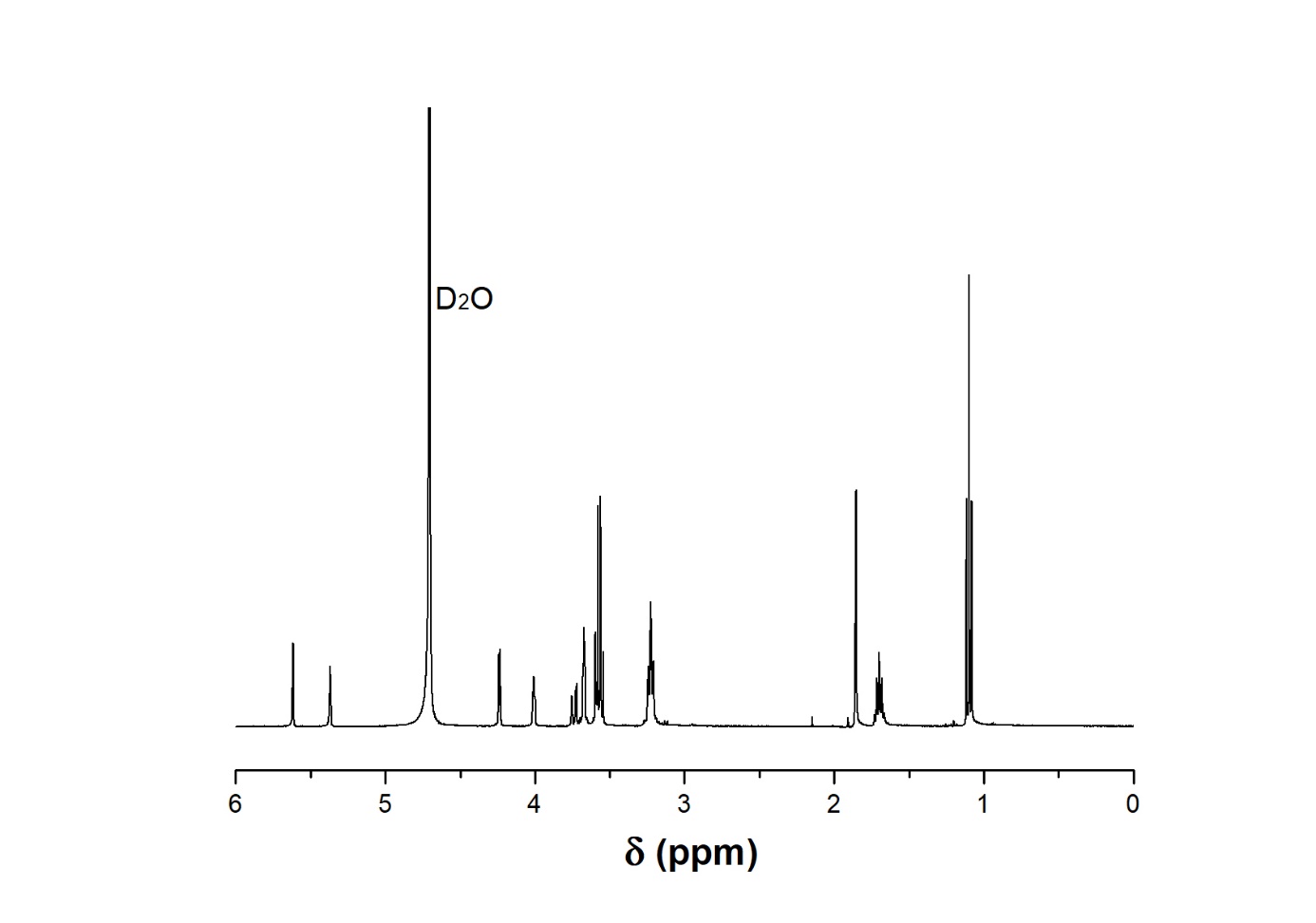


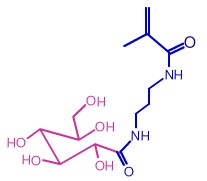


**GAPMA**

**Figure S2.** ^1^H NMR spectra for 3-gluconamidopropyl methacrylamide (GAPMA) monomer in D_2_O.


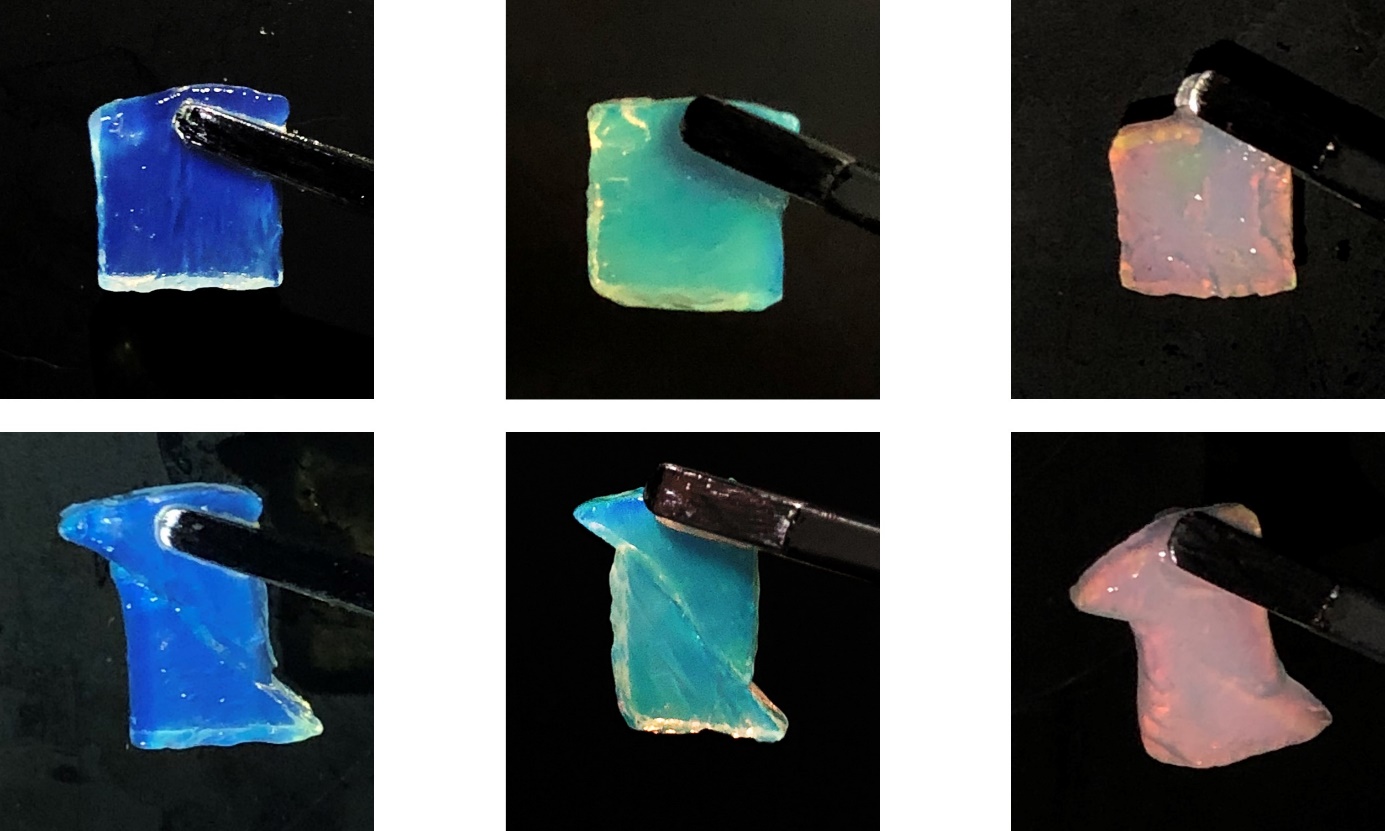


**Figure S3**. The enlarged images of self-healing process of the colloidal PC hydrogels.


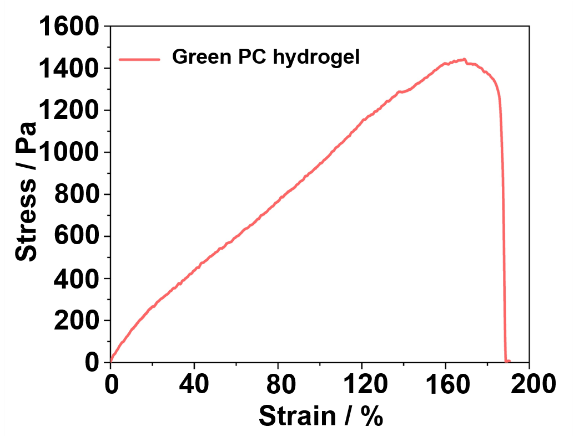


**Fig. S4.** The stress-strain curve of tensile test of green colloidal PC hydrogel.


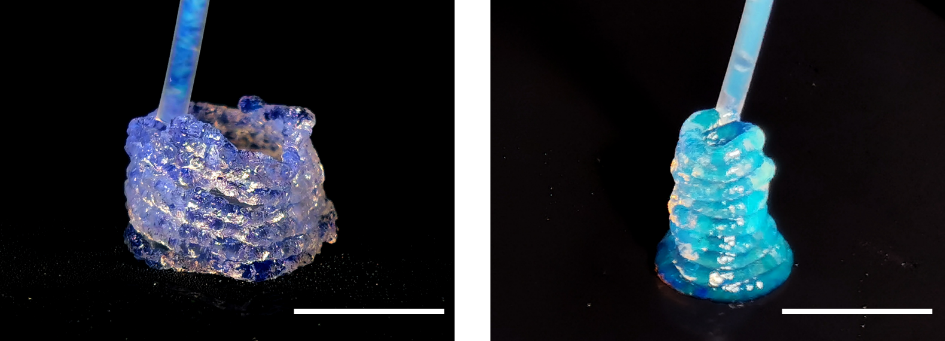


**Fig. S5.** 3D printability of the blue and green PC hydrogels (scale bar = 5 mm).
